# On Contagion

**Published:** 1825-06

**Authors:** Charles Larkin


ART. II.—*On Contagion.* By CHARLES LARKIN, Esq.

[Concluded from page 364.]

I now advance to the concluding portion of my paper on Contagion. Whoever has done me the favour of dispassionately perusing the preceding parts, if he do not go along with me to the full extent of the conclusion which I draw, must at least acknowledge that contagion has very much less to do with the production, propagation, and continuance of pestilence, than he was previously inclined to suppose that it had; and that plague owes its origin to a cause that has hitherto completely eluded detection,—continues as long as, and no longer than, that cause continues in operation,—and disappears as soon as it ceases to shed its deleterious influence. Its beginning and end are both involved in darkness and mystery. Thus far the most cautious and timorous induction will lead: thus far the most timid of reasoners, after the nicest and most scrupulous balancing of antithetical facts and arguments, must go. But he, who, held back by no vain terrors, and impeded by no sturdy prejudices, but animated by a pure love of truth, boldly and vigorously pursues facts to the legitimate consequences which they point to, will coincide in the opinion that contagion is a fable, that, like many other monstrous fictions of the human mind, which has in all ages teemed with "Gorgons, Hydras, and Chimeras dire," has had sufficient terror in its sound to make the world turn pale, but has no real and substantial existence. It is one of those shadowy and dreadful phantoms which people the world of imagination, and which, though unreal mockeries, exercise a horrible tyranny over the souls of men. Those who boggle at this conclusion, and have not sufficient strength of mind to scorn and reject the vain and idle stories (idler than the vainest of nursery-tales,) on which this doctrine is bottomed, are certainly to be commiserated; for they keep themselves in perpetual agitation, and propagate to others the same unfounded terrors that disquiet their own bosoms.

As to the doctrine promulgated by Sir A. B. Faulkner, Dr. Granville, and the committee of the House of Commons, that plague is communicated by contact alone, it must be confessed that it lies, like Enceladus under Etna, crushed and buried beneath that mountain of undenied and undeniable facts that I have heaved upon it. The number of these facts I could have easily increased, and, had it been necessary, have piled Pelion upon Ossa: but the labour would have been a mere waste of time and strength; for the doctrine is in itself so improbable, that it carries its own refutation along with it; and those who support it I regard in no other light than that of mere alarmists.

Who, then, can review with a cool and dispassionate eye, and reflect upon the incontrovertible facts which have been produced, without an intimate conviction, (and it is one, like all truth, full of consolation,) that plague is, both in its origin and continuance, independent of contagion? We have beheld instances of individuals leaving infected towns, and falling ill of the plague in the places to which they emigrated, without communicating the disease to any member of the family in which they lodged, or extending it to any other inhabitant of the town. We have seen villages ravaged by pestilence, whilst contiguous villages, commercing with them, were free from its visitation. We have beheld pestilence devastating the vicinity of Aleppo, without entering within the walls of that populous town, though the inhabitants did not commit the folly of throwing a circumvallation around, as if an enemy had actually sat down to besiege, it; nor did they plant a guard of soldiers at every gate, to prevent all egress or ingress. We have seen Scio in constant communication with Smyrna, Alexandria with Constantinople, and Aleppo with Egypt, Constantinople, and Smyrna, during the very height and utmost rage of pestilence, without any extension of the disease to these places, although no precautionary measures were adopted. We have beheld it arising from causes beyond human divination, and declining with rapidity, without the aid of human intervention. We have seen it overleaping the mounds and barriers that have been erected to hold it at a distance, proceeding unimpeded in its course, and breaking, as though they were but spiders' threads, all the interruptions which mortal ingenuity could throw in its way, rushing onward with the violence and swiftness of a whirlwind, and, after desolating every place in its progress, and after leaving the diminished numbers of mankind as a monument of its power, suddenly disappearing from the earth; and we have seen it disappear at that precise time when all restriction on human intercourse was taken off,—when all confessed the vanity of any attempt to arrest it,—when the proudest were subdued into submission,—when every eye was raised to Heaven, as to their last and only hope and expectation, and every heart acknowledged, how feeble was the resistance of a thousand mortal arms, when arrayed against the single arm of the Omnipotent.

It is impossible to contemplate these facts, and resist the conviction that contagion is a misapprehension and falsehood. Contagion and these facts (the truth of which we have the contagionists themselves confessing,) cannot co-exist. If contagion be true, these facts are false: if these facts be false, then is experience a cheat, and fact a liar.

But it may be said, that the same disease may vary in its character, and evince contagious properties at one time, and not at another. This is a charge against the constancy and uniformity of nature, which all that we see proclaims unfounded. If nature were variable, experience would have no foundation to rest upon, and Philosophy, which is the daughter of Reason and Experience, could not exist. Unless the past were the type and shadow of the future, reason would be but a blind guide, and memory but a lumber-room of useless facts and unprofitable observations. The investiture of the mind with all that array of faculties which adorn it, and which, under the present constitution of the universe, it has made so splendid an exercise of, would have been a cruel mockery, and a dooming of all mankind to the fate of Tantalus. Like the fabled waters in which he is represented to be steeped, and which ever fly the approach of his dry and parched lips, things would have flowed on in their eternally diversified and irregular course, and ever have escaped from the grasp of the mind. I would rather, therefore, believe that the contagionists were bad observers, than give them the credit of having discovered an irregularity in the course of nature, or one single instance of tyranny and caprice in the arrangements of Providence.

The rapidity with which plague is propagated in certain situations, its confinement to these situations, and the general immunity of those who are situated at a distance from them, have little or no intercourse with them, and breathe a purer atmosphere, have given origin to the notion of contagion. But we always observe that men are healthy, and enabled to resist the encroachments of disease, in proportion to the salubrity of the situation in which they live, the purity of the atmosphere which they breathe, and the roominess of the apartments in which they dwell. The more foul and stagnant the atmosphere, the more narrow and unventilated the cells, and the more crowded the dwellings of men, the more prone they are to disease, and the more easily they fall a prey to the emissaries of death. We, therefore, always find epidemics to rage with the greatest violence in the filthy alleys they inhabit, and the populous hives of the poor; and that they by no means spread with equal violence and celerity in situations through which the air can sweep with a free and unobstructed course. The rules of segregation and cleanliness which the Franks observe, though they carry them to an unnecessary and ridiculous extreme, are in themselves wholesome, as they tend to prevent that deterioration of the air, which must always occur when multitudes of people are congregated together in unventilated apartments; and, consequently, by preserving the air as pure and uncontaminated as possible, they are enabled to contend against the influence of those causes which produce and dispose to disease. The situations, also, which they choose to live in, when they are enabled to do it, are airy and spacious,—the delightful villages on the Bosphorus, &c. and the squares and open streets of cities. Dr. Russel tells us that he frequently examined the sores of patients within four feet; yet neither he, nor any of his family, or any other inhabitant of the *square* in which he lived, were infected by the contagion of such a number of pestilential patients. Yet all these precautions do not produce absolute security; for the instances are not rare of those who, without any communication with the sick, fall victims to the disease, notwithstanding that they strictly observe, not only all the precautions which wisdom dictates and experience sanctions, but those which folly and terror have superadded.

Before I proceed further, I must do away with an objection that has been brought *in limine* against the doctrine of which I am the feeble, but sincere, advocate. It is said by some of those who are continually ringing the alarm-bell in the ears of the public, that, to disseminate among the people the notion that contagion does not exist, would occasion a supine submission to the evil, which would be most dangerous and destructive to the community. There is reason to believe that the fact is in favour of the contrary supposition. We have no reason to believe that the mortality is greater among the Turks than it has been in European cities visited by pestilence. On the contrary, it has been asserted that the mortality is less. In Turkey, one great disposing cause is absent: I mean the terror which is universal where this doctrine of contagion prevails. All physicians, says the learned Dr. Mead, agree that fear, despair, and dejection of spirits, dispose the body to receive contagion, *and give it a great power* when it is received. This is an observation which gives great countenance to the foregoing supposition. But the doctrine of the non-existence of contagion has another great advantage: it secures attendance to the sick, and thus has a tendency to diminish the mortality; for none are left, where it prevails, to die from neglect. It is to the eternal praise of the Turks,—and one is pleased to observe the harsh and ferocious features of their character softened by this trait of magnanimous humanity,—that they expose themselves, cheerfully and without fear, to whatever danger there may be in the performance of their duty, are unremitting in their attention to the sick, and, if they do fall martyrs to the disease in consequence of their charitable assiduities, they fall with the pleasing reflection that they have not been guilty of a base and cowardly dereliction of their duty; and are not haunted by that remorse which will inevitably fasten, like a vulture, on that man's heart who has been deaf to the call of nature, of honour, and of humanity. Among them, the barbarity of deserting the sick has no place; and, in this respect at least, the Crescent beams more gloriously, and seems exalted above the Cross.

Speaking of contagion, Gibbon says, "While philosophers believe and tremble, it is singular that the existence of a real danger should have been denied by a people prone to vain and imaginary terrors; yet the fellow citizens of Procopius were satisfied, by some short and partial experience, that the infection could not be gained by the closest conversation; and this persuasion might support the assiduity of friends and physicians in the care of the sick, whom inhuman prudence would have condemned to solitude and despair." Even, then, were the doctrine of the non-existence of contagion as false as, on my soul, I believe it to be true, still I assert that it is a beneficial falsehood; a delusion which a wise and good man should do his best to propagate; for, while it would do away with an unnecessary terror, it would make the false friend and the faint-hearted physician, who is ever ready to put pinions to his heels, and lead the flight, at the first sound and alarm of danger,—even these would it make bold and fearless in the discharge of that duty, which the one owes to his connexions, and the other to society.

In spite of all our precautions, though we make them a thousand times more rigid than they are, and complete and perfect our system of quarantine, plague may come. Do not entertain so weak and silly an imagination, as that it is in the power of man to prevent it; and if it should come, and the doctrine of contagion should continue to prevail, (which God avert!) the horrors, the aggravated horrors of a time, fearful enough in its own nature, but rendered a thousand times more fearful by this horrid concomitant,—what tongue can tell, or what heart can conceive! While yet upon the threshold, I recoil and shrink back from my task, overpowered by the sense of my own weakness and inability to do it justice. Would that I had the Atlantean shoulders fit for the support of so mighty a subject!

I have, then, to show that the doctrine of contagion is productive of misery and crime. This I shall do chiefly by referring to the history of the last plague, which filled all England with consternation and mourning; because, by so doing, I hope to bring the question home to the business and bosoms of men. No sooner, then, had the plague declared itself, than the terrified inhabitants,—at least, all who had the means of so doing,—crowded with precipitation to the door of the lord-mayor's house, demanding certificates of health, without which they could neither pass the lines, nor gain admittance into any of the provincial towns. Every family was busy in preparations for their departure, and every road leading from this immense metropolis was choked up with these fugitives from their homes and their duties. So great was their number, that many of the largest and most-frequented streets presented a most dreary aspect, every house being shut up, so that the eye looked around in vain for any signs of the busy cheerfulness of life. Not a human form was to be seen moving about, with the exception of here and there a solitary watchman, who, instead of relieving, rather heightened the dismal character of the scene. The hum of men, and the tread of multitudes, were no longer to be heard; but, in their place, silence reigned, and solitude. It was the boast of the ferocious Attila, that the grass never grew where his horse had trod: the doctrine of contagion (a word, in whose sound there is as much terror as ever there was in the name of that fell destroyer of his kind,) produced the very opposite effect, and occasioned the prodigious spectacle to be seen of grass growing in the streets of London.

Not only did these people run away from the danger, but they took the inhuman precaution of discharging all unnecessary attendants. Vanity laid aside her pomp, and pride was terrified into humility. Rank dispensed with her equipage, and, instead of flying through the country with a numerous retinue at her heels, assumed an appearance of plainness and simplicity. This was a step, to the adoption of which she was forced by fear; for, at this dismal period, nothing was so much dreaded by man as his fellow man. Every one eyed his neighbour askance, and glared upon him with a fearful look of suspicion. The human face divine was no longer an object, the sight of which was to be longed for, and the deprivation of which Milton, in a beautiful and affecting passage, has deplored as the worst evil of blindness. It is one of the mischiefs of this doctrine, that it totally alters the character, and depraves the habits, of the noblest creature that walks beneath the sun. It robs him of the distinctive character of his race: he remains no longer a gregarious and social being, but roams about, like a savage beast, gloomy and solitary, impelled by no other feelings than his own wants; all the generous and exalted sentiments of his nature being absorbed, and sunk in that great virtue which this doctrine inculcates—a selfish regard to individual safety. Not only did pride and rank consent to be shorn of their beams, and to diminish their train of attendants, but the merchant, the manufacturer, and tradesman, of every kind, dismissed all their labourers and workmen. In fact, every trade, of every kind, except such as related to immediate subsistence, was at a dead stop. To state this in broad and general terms, is to give my readers no idea of the variety and compass of the misery which this determination occasioned; but to go into detail I have not room. Suffice it then to say, that the whole of the labouring population, servants (male and female), mechanics, and labourers, were deprived of the means of subsistence. It will be wiser in me to leave to the general conception of my readers the miserable condition of this innumerable multitude of hard-working and laborious men, exposed by the humanity of their masters to the double chance of death from the plague of hunger and from pestilence, with no other dependence than private charity, for their strength, the gift of Providence, was converted by Man into a useless possession, with no place of shelter, and none for repose, but the bare ground or open field, a ruined barn or shed, which they shared with the beast of the field. For it was another of the miseries of this exigent time, that, every feeling of benevolence and hospitality having been eradicated from the breast by this doctrine, the poor outcast from society could not even extort from Avarice that asylum which he looked for in vain from Charity. We have it upon record, that numbers were turned out of their lodgings, and, being once exiled from their homes, they could find no other. Every wanderer was looked upon as an infected person, and as such was sternly refused admission at every door. The consequence was, that these unfortunate and rejected beings died, like folds of sheep, in the fields and on the road-side, without assistance and without compassion. They fell before the disease, like grass before the mower's scythe. So great was the mortality among them, that we are told by Dr. Hodges it was emphatically called the Poors' Plague.

But it is upon the condition of the sick that I wish principally to fix the eye. I have pointed out what it is in countries where this notion of contagion does not prevail: look now upon the reverse of that picture.

As soon, then, as any one is so unfortunate as to fall sick of this most terrible of all diseases, it is the signal for his friends to desert him. As this doctrine teaches them that there is death in his very breath and touch, instead of becoming an object of solicitude and care, he becomes one of horror and aversion. What imagination can picture to itself a condition more dreadful and horrible than that of a man, who, when overtaken by a mortal distemper, which is shortly to break all the ties that bind him to the earth, when tortured by pain, and lacerated by all the agonies of extreme terror, finds himself abandoned at the very moment at which the attentions and solicitudes of friends and children is most soothing and consolatory to the soul, who sees neither help on earth nor hope in heaven, and finds himself as completely torn from the bosom of society by disease, as he could by possibility be by death? A person in this condition is thus feelingly apostrophised by the poet Quarles, who had seen and shuddered at the horrors which he describes:—"How is the bitterness of thy death multiplied by the quality of thy fears! Were it a sickness whose distraction took not away thy means of preparation, it were an easy calamity! Were it a sickness whose contagion dissolved not the comfortable bonds of sweet society, it were but half a misery! But, as it is,—sudden, solitary, and incurable, what so terrible,—what so comfortless. At such times,

> Heaven's music, which is order, seems unstrung;

> And this brave world,

> The mystery of God, unbeatified,

> Disordered, marred, where such strange scenes are acted."

At this dreadful hour, his distemper is alleviated by the warm and gentle hand of friendship, and the soul of the Turk is breathed out in the arms of affection: but the Christian's home is desolate, and he, poor wretch! dies with the most sad and dismal of impressions that he is a forsaken man, and that "the world and the world's law are both against him." Or, if he has attendants, who are they? Convicts and felons. But the contagionist shall describe the attendants whom he provides for the sick:—"It is no small part of the misery," says Dr. Mead, "that attends this terrible enemy of mankind, that, whereas moderate calamities open the hearts of men to compassion and tenderness, this greatest of evils is found to have the contrary effect, and barbarities are practised unknown at other times. Accordingly, Diemorbrock informs us that he himself had often seen these hospitals committed to the charge of villains, whose inhumanity has suffered great numbers to perish by neglect, and that sometimes they have even smothered such as have been very weak, or have had nauseous ulcers difficult to cure. Insomuch that, in many places, the sick have chose to lay themselves in fields, in the open air, under the slightest coverings, rather than to fall under the barbarous hands of those who had the management of these hospitals."

Exactly similar enormities occurred during the plague of London. Children forsook, and were forsaken by, their parents. Maternal tenderness, which is rather an instinct of nature than a virtue, became so rare and uncommon as to be celebrated, in the instances in which it was displayed, as heroic virtue. Deserted by the attendants whom Nature appointed them, the sick were left to the mercy of hags, who were possessed rather with the spirits of fiends than human souls: a perfect race of Canidias, who watched with eager and demoniac eyes for the moment of dissolution, that they might glut their rapacity with plunder; and, as if the progress of a quick distemper was too slow for their hungry and impatient passions, there they stood, with the poison-bowl in one hand and a halter in the other, ready to strangle or to poison the helpless and forsaken wretch.

Such is the humane effect which this doctrine produces upon the minds of men! It has been said by a great master of irony, that it is a preposterous method to judge of the truth of any doctrine by its apparent consequences; but it was said when he wished to place the absurdity and falsehood of certain paradoxical opinions in the strongest and intensest light. And, surely, it cannot be contrary to reason to argue that a doctrine, which has made thick the blood of man, and stopped up the access and passage to remorse,—which has caused the wretched to be forsaken in the hour of their direst necessity, and most urgent distress,—which has scattered misery and despair far and wide, and deprived even the parturient woman of assistance, (for it is upon record that, at these times, the woman in labour has shrieked for help in vain;)—surely, I say, it is not contrary to reason to argue that such a doctrine is not only false, but approaching in its nature to blasphemy, inasmuch as it arraigns the benevolence of God; which divine attribute is not less conspicuous in all his works, than his marvellous wisdom and infinite power.

I have next to draw the attention of my reader to the mode in which the infected were shut up; and in all that I shall have to say on this subject, I shall be borne out by the advocates and champions of contagion. All my assertions I have proved from their works, which have been my text-book; for, possessing not a shadow of influence, natural or adventitious, all that I have said, were it not so enforced, would pass for as little as the ravings of the idle wind. "The methods," says Dr. Mead, "used on such occasions have always had the appearance of a severe discipline, and even punishment, rather than of a compassionate care. As soon as any house was infected, it was kept shut up, with a large red cross, and these words, 'Lord, have mercy on us!' (and well might they cry on the Lord for mercy, for from man they found none,) painted on the door. Watchmen attended day and night, to prevent any one's going in or out, except such as were allowed by authority; and this continued at least a month after all the family was dead or recovered. It is not easy to conceive a more dismal scene of misery than this. Families locked up from all their acquaintance, though seized with a distemper which, the most of any in the world, requires comfort and assistance; abandoned to the treatment of an inhuman nurse; and strangers to every thing but the melancholy sight of the progress death makes among themselves;—with small hopes of life left to the survivors, and those mixed with anxiety and doubt, whether it be not better to die than to prolong a miserable being after the loss of their best friends and nearest relations. Nothing can justify such cruelty, but the plea that it is for the good of the whole community, and prevents the spreading of the infection." The humane mind of Dr. Mead revolts and is horror-struck at these enormities, which, in his opinion, increase the mischief they were intended to prevent; and he recommends a less severe and less cruel mode of treatment. But what security, I ask, should we be again visited by this dreadful scourge, have we that similar enormities will not again be perpetrated?—enormities, at which the face of heaven doth glow with horror and indignation, and every mind, that has within it the slightest scintillation of humanity, is perfectly thought-sick.

Dr. Hodges has also made some very pertinent remarks on this subject. "The consternation of those who were thus separated from all society, unless with the infected, was inexpressible; and the dismal apprehensions they laboured under made them an easier prey to the devouring enemy. And this seclusion was, on this account, the more intolerable, because, if a fresh person was seized in the same house but a day before another had finished the quarantine, it was to be performed over again, which caused such tedious confinements of sick and well together, as sometimes caused the loss of the whole. Moreover, this shutting up infected houses made the neighbours fly from theirs, who otherwise might have been a help to them on many accounts; and I verily believe that many who were lost might have been now alive, had not the tragical mark upon their doors drove proper assistances from them." The indignant feelings which must boil in every manly breast on reading these passages, are a better comment than any I can make. Every life that was lost is a terrible denunciation of the practice, and the doctrine on which it was founded.

We who are at liberty, and indulge in speculation while we view the evil at a distance, may talk in the most complacent style imaginable of a six-weeks or three-months' confinement; but the reality is no such light thing. Why, we scarce restrain our indignation at the tyrant, who, at the sound of the curfew-bell, ordered every light and every fire to be extinguished. There is something in liberty so dear to the heart of man, that the soul kindles at the slightest infringement of it. Those who may have been confined by an attack of illness to their homes, where they may have been able to relieve the tediousness of confinement by the converse of friends, must still have felt, even with this lenitive, with how heavy and languid a motion the foot of time dropt, and to the invention of what expedients they were driven to unload the mind of that most intolerable of all sensations, the listlessness of inaction and languor of perfect repose:—what, then, must the sickness of their hearts have been, whose hopes of enlargement were liable to be so cruelly deferred, and whose confinement might be so indefinitely prolonged, and who, instead of regarding society as a solace and relief, were taught by their terrors to look upon the company of their fellow-prisoners as the very worst evil of their imprisonment. A regard to his individual safety may require the confinement of a madman,—the justice due to an injured man, the incarceration of a criminal; but that a sane and innocent man should be put into circumscription and confine, because he has been overtaken by sickness, or because he did not pass by, but lavished his attention upon those whom the voice of Nature, never to be disregarded with impunity, loudly called upon him not to desert,—because he did not feel a sick man's pulse through a tobacco-leaf,—or because he had accidentally touched something *supposed* to be infected, is a tyranny which I have no language sufficiently strong to express my soul's hate of; a cruelty, which nothing but the supremest necessity, and that clearly evinced, could justify or palliate, and which, even under such circumstances, the mind would suspend its judgment of, rather than absolutely approve. But in this case, when done merely to appease the terror excited by a doctrine, which is as baseless as the fabric of a vision, and which bears no more resemblance unto truth than villany does to honesty, and which carries upon its harsh and haggard features the very stamp and impress of barbarism, the mind cannot regard it in any other light than an act of the most hateful tyranny, or be actuated by any other feelings than the perpetration of the deepest injustice was calculated to engender.

2

But what shall we think of the moral effects of this doctrine, whose supporters, like some other people of notorious memory, have always "douce humanité" on their tongues, while every action of their lives is giving the lie to their professions, which, after screwing the human mind up to the highest pitch of terror, has induced those who act upon the belief that it is true, to shut up the terrified wretch in the same place with the object of his utmost terrors. We read with detestation of the monster who flung unoffending men into a lion's den,—who bound together a living body and a putrid carcase, and left the affrighted wretch to tug his life away in convulsive endeavours to extricate himself from the foul embrace of Death: shall we regard with feelings of less abhorrence the conduct of those Christian magistrates who encaged a healthy man with a pestilent being, whom they had taught him to avoid as an object of the utmost aversion, and most unutterable horror? I despair of being able to convey even a faint idea of the horrors of such a situation to such a man. I shall not, therefore, make the attempt; but I can recount the consequences which resulted from this unfeeling treatment. In many instances, furious despair and raging madness; some died through fright; others were terrified into self-destruction; others fell into a state of melancholy, from which they never recovered; and others, deprived of Heaven's best gift, their reason, became idiots for life. Mothers, robbed of a mother's heart, imbrued their hands in the blood of their children; others, in a wild delirium, broke loose from their confinement, and, like enraged bulls from the slaughter-house, hunted by a pack of butchers, ran through the town; while others murdered their guards, and thus made their escape. Sights so strange and deform as these who can behold, and refrain from venting curses, both loud and deep, upon the monster Contagion, whose brood they are?

And on what plea are all these abominations justified? On the tyrant's plea, necessity, and the safety of the people. Away with such hollow and miserable pretences. A kind attention to the sick I cannot but consider as a great moral duty, binding upon every man, in all times and in all seasons, and paramount to every consideration, both of a public and private nature. By the highest authority that ever appeared on the earth, the torment of everlasting fire has been denounced against him who visiteth not the sick man and the prisoner. The law is universal, and without exception. The man who has fulfilled all his duties to society has no right to be selected, and offered up as a victim to free his fellows from an imaginary danger. The devoted wife, the affectionate husband, the anxious parent, the dutiful son, and the faithful servant, have no right to be considered as the very καθαρματα, the offscouring and recrement of society, and immolated as a kind of expiatory sacrifice, to propitiate the wrath of Heaven, or allay the terrors generated by this doctrine. Should, then, a doctrine, (to speak of it in the most lenient terms,) of which there are strong reasons to doubt the truth, which tends to shake to its very centre the whole order of society, and to loosen the eternal foundations of morality itself, be permitted to exist a moment unstrangled, unless it were established on testimony ten times more irrefragable and convincing than any other truth? The contagionists work upon our fears, and address themselves to the basest portion of our nature. Incapable of carrying conviction home to any breast, they can terrify those by their tales whom their reasonings cannot satisfy. This is the vantage ground, which they have long possessed, but from which I do trust that, by the vigorous attacks and perseverance of their opponents, they will be quickly driven. With ignorance, fear will be expelled;—abolish the fear that prevails, and the doctrine will only be remembered with abhorrence; and the tales and arguments on which it has been founded will meet with the contempt they deserve.

My notion of morality I would rather learn at the feet of some of the great sages of antiquity, than in the modern schools of ethics. They were men whose mental eye penetrated far deeper into the nature of things, and saw far more solid supports and buttresses for morality to repose upon, than any wretched and temporary considerations of private safety or public expediency; and I proudly place in contrast with the doctrines of our modern ethical philosophers the following sublime notions, drawn from a half-recovered work of a great orator, statesman, and moralist:—"Est quidem vero lex, recta ratio, naturæ congruens, diffusa in omnes, constans, sempiterna, quæ vocet ad officium jubendo, vetando a fraude deterreat, quæ tamen neque probos frustra jubet, aut vetat, nec improbos jubendo, aut vetando movet. Huic legi nec abrogari fas est, neque derogari ex hac aliquid licet, neque tota abrogari potest. *Nec vero aut per Senatum, aut per populum solvi hac lege possumus.* Neque est quærendus explanator, aut interpres ejus alius; nec erit alia lex Romæ, alia Athenis, alia nunc, alia post hac; sed et omnes gentes, et omni tempore una lex, et sempiterna, et immortalis continebit; unusque erit communis quasi magister et imperator omnium Deus ille, legis hujus inventor, disceptator, lator; *cui qui non parebit, ipse se fugiet, ac naturam, hominis aspernabitur, atque hoc ipso luet maximas pœnas, etiamsi cetera supplicia, qua putantur, effugerit.*"

I argue, then, against this doctrine of contagion, because it leads to the infraction of this one, sempiternal, and immortal law, which enfolds in its vast embrace all nations and all times,—which admits neither of a partial or total repeal, and from the observance of which neither parliaments, nor kings, nor people, can absolve,—by the terror which it produces; for there is more terror in its very sound than ever there was in the name of Goth, of Hun, or of Vandal; and all the woes and miseries which these barbarians have brought upon the earth, have been mercy to the wild havoc it has occasioned. It has separated wife from husband, child from parent, and withered those instinctive affections of the soul which bind the mother to her offspring. It has made populous places solitary: not only has it planted solitariness where before there was society, but it has scathed and made waste the human heart, scorching and blasting all the best and most amiable feelings of our nature. All that is divine in the composition of man it has sublimed and driven off, and left behind the mere earthy residuum, the *caput mortuum* of mortality. It has riven asunder all the ties that hold the moral elements of the world together, and which, while they are "light as air," should be strong as cramps of iron. In arguing against this doctrine, which reverses the parable of the good Samaritan, and, applauding the priest and the Levite, teaches that the sick and helpless should be neglected and passed by, and our cares expended on the vigorous and healthy, I consider myself as the advocate of humanity; for from the loins of this monster have all these evils sprung.

